# Determination of site-specific carotid-intima media thickness: common –carotid artery and carotid bifurcation in hypercholesterolemia patients

**DOI:** 10.12669/pjms.295.3830

**Published:** 2013

**Authors:** Samia Perwaiz Khan, Pashmina Gul, Saleem Khemani, Zia Yaqub

**Affiliations:** 1 Dr. Samia Perwaiz Khan, MBBS, M. Phil, PhD, Associate Professor of Pharmacology, Ziauddin University, Karachi, Pakistan.; 2 Dr. Pashmina Gul, FCPS, Dr. Ziauddin Hospital, Karachi, Pakistan.; 3 Dr. Saleem Khemani, MCPS, DMRD, Dr. Ziauddin Hospital, Karachi, Pakistan.; 4 Prof. Dr. Zia Yaqub, DABIM, DABCVD, Consultant Cardiologist, National Institute of Cardiovascular Diseases, Karachi, Pakistan.

**Keywords:** CIMT- Carotid- intima media thickness, CCA- Common carotid artery, BULB – Bifurcation of carotid artery, B-mode ultrasound

## Abstract

***Objective: ***To determine site specific carotid intima-media thickness: common–carotid artery and carotid bifurcation in hypercholesterolemia patients as a marker for atherosclerosis.

***Methods:*** Fifty patients with hypercholesterolemia and twenty controls were selected after getting informed consent regarding the investigation of carotid- intima media thickness by B-mode ultrasound. All the patients of hypercholesterolemia with LDL-C > 160mg/dL had family history of coronary artery diseases. This procedure was carried out in the Radiology Department of Dr. Ziauddin Hospitals. Measurement of carotid -intima media thickness, B-mode ultrasonography of common carotid artery, carotid bifurcation and internal carotid artery (left and right carotid arteries) was performed with Toshiba (M# SSA-580A/E2) ultrasound scanner with linear probe. The posterior or far wall of the carotid artery is, the distance between the leading edge first bright line (lumen -intima interface) and the leading edge of the second bright line (media-adventitia interface) of far wall was recorded as intima -media thickness. The average mean of six segments of intima-media thickness was taken as mean CIMT of right and left common carotid, bifurcation and internal carotid arteries.

***Results: ***Maximal CIMT was significantly increased at sites common carotid, carotid bifurcation and internal carotid arteries in fifty patients with hypercholesterolemia as compared to controls. At carotid bifurcation mean of maximal CIMT was (0.9+ 0.3mm). Range of maximum CIMT in hypercholesterolemia patients was (0.8- 3.3mm) and in controls (0.4- 0.8 mm). The thickness was more frequently increased at site of bifurcation.

***Conclusions: ***Carotid intima- media thickness in hypercholesterolemia patients was increased and carotid bifurcation was site that has shown greater increase in intima-media thickness and plaques in these patients predict high risk for atherosclerosis.

## INTRODUCTION

Risk factors contribute to atherosclerosis through gradual arterial changes that may produce ischemia by either progressive luminal narrowing or more commonly, by sudden plaque rupture or intimal erosions with formation of an in situ occlusive thrombus.^1^

A widely accepted, convenient marker of atherosclerosis is carotid artery intima-media thickness (IMT).^2^ Variation in the progression of atherosclerosis at different arterial sites, and not error in ultrasound measurements, is thought to contribute to some discrepancies in the prediction of coronary events.^3^ The use of mean maximum CIMT rather than mean common CIMT as the primary outcome measure in Randomized Control Trials designed to evaluate the efficacy of pharmacological and non- pharmacological interventions in carotid artery atherosclerosis.^4^

Atherosclerotic changes can be measured by carotid intima-media thickness by B-mode ultrasound is surrogate end point in cardiovascular outcomes in clinical trials.^5^ Carotid intima-media thickness has been measured to determine risk of atherosclerosis.^6^^-^^12^ Studies have shown that increased carotid intima-media thickness is high risk for cardiovascular diseases.^13^^-^^15^ B-mode ultrasound imaging (Carotid Doppler) can visualize noninvasively the superficial arteries in every stage of arthrosclerosis. As observed by de Groot et al an average healthy person will have IMT of 0.78mm at 76 years of age while FH patient has this CIMT at 40 years of age.^15^ CIMT assessment used in randomized control trials to determine effects of therapy.^16 ^ CIMT can be used as a surrogate end point for intervention trials.

The aim of this study was to determine carotid intima- media thickness at different sites, common carotid artery and carotid bifurcation in hypercholesterolemia patients as marker for atherosclerosis.

## METHODS

Fifty cases with hyperlipidemia were from National Institute of Cardiovascular Diseases and Dr. Ziauddin Hospitals and twenty controls were selected after getting informed consent regarding the investigation of carotid- intima media thickness by B-mode ultrasound. Age range of cases and controls was 22 to 60 years. All the patients of hyperlipidemia with LDL-C >160mg/dL (lipid profile done after 12 hours fasting) had family history of coronary artery diseases. This study was approved by Ethical Committee of Ziauddin University.

***Sites for measurement of Carotid Intima- Media Thickness: ***This procedure was carried out in the Radiology Department of Dr. Ziauddin Hospitals. Measurement of carotid -intima media thickness, B-mode ultrasonography of common carotid artery, carotid bifurcation and internal carotid artery (left and right carotid arteries) was performed with Toshiba (M# SSA-580A/E2) ultrasound scanner with linear probe. The posterior or far wall of the carotid artery is, the distance between the leading edge first bright line (lumen-intima interface) and the leading edge of the second bright line (media-adventitia interface) of far wall was recorded as intima -media thickness.

The average mean of six segments of intima-media thickness was taken as mean CIMT. Wall thickness was defined as the distance from the leading edge of lumen-intima thickness interface of the far wall of leading edge of media-adventitia interface of far wall.^17^ Frozen images of these segments were of carotid intima- media thickness mean and maximum values were calculated by the computer.

Assessment of CIMT at multiple sites determines frequent plaque thickening that are common in carotid bifurcation in patients with high levels of total cholesterol and LDL-Cholesterol. 

***Statistical analysis: ***Comparison was done with mean and maximum of CIMT of hypercholesterolemia patients and controls. For carotid intima- media thickness the mean and maximum values were calculate by applying student’s t-test for right and left mean IMT of common carotid, bifurcation and internal carotid arteries. Using student’s t-test for comparison of quantitative data (CIMT thickness) compared between hypercholesterolemia patients and controls (Table-I).

**Table-I:** Comparison of CIMT in hypercholesterolemia patients and controls

*Maximal CIMT (mm)*	*Hypercholesterolemia (n=50)*	*Controls (n=20)*	*p*
	*Mean ± SE*		
Common carotid	0.85± 0.26	0 .65±.02	0.001 **
Bifurcation	0.9± 0.3	0.7±.02	0.001**
Internal Carotid A	0.75± 0.44	0.5±.05	0.001**

Highly significant- p-value 0.001**

## RESULTS

Images of the segments of carotid intima-media thickness of common carotid, carotid bifurcation and internal carotid arteries were taken by B- mode. Mean of maximum values were calculated by the computer. Assessment of CIMT at multiple sites determines frequent plaque thickening that are common in carotid bifurcation and internal carotid artery in patients with risk of cardiovascular diseases. It is a reproducible study assessment was done by two observers. Plaque seen mostly at bifurcation were reveled in eleven cases with familial hypercholesterolemia.

**Figure:**
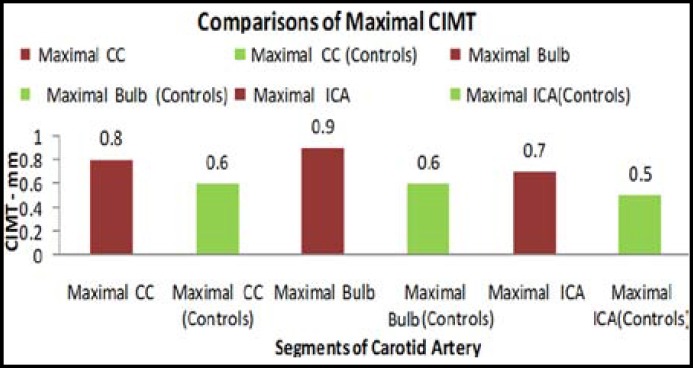


Maximal CIMT was significantly increased in subjects with hypercholesterolemia as given in Table-I. CIMT comparison of hypercholestrolemics and controls by students t-test (p<0.001***). Range of maximum CIMT in hypercholesterolemia patients was (0.8-3.3mm) and in normolipidemic controls (0.4- 0.8 mm) (Table-I). The thickness was more frequently increased at bulb in patients with hypercholesterolemia. CIMT of hypercholesterolemia patients at sites, common carotid (Fig.1-A) and carotid bifurcation as shown (Fig.1-B).

## DISCUSSION

The present study shows increased Carotid intima-media thickness in patients with FH and most of these patients have shown to have developed early coronary and cerebrovascular diseases. 

CIMT should be measured preferably on the far wall^18^ values from the near wall are less reliable. It is possible to visualize plaques in the artery with ultrasound technique. Plaque is a focal structure encroaching into the arterial lumen.

In clinic an CIMT >0.9 mm or presence of plaque may support a more intensive risk factor and intervention at individual level.^19^ Bifurcation was found to have the highest correlation with CIMT. If a single ultrasound is to be used, bifurcation should be the preferred site.^20^

This study shows an increase in mean carotid intima-media thickness more in area of bifurcation. Also calcified plaques were seen in patients with very high LDL-cholesterol levels (Fig.1.B).

**Fig.1(A):**
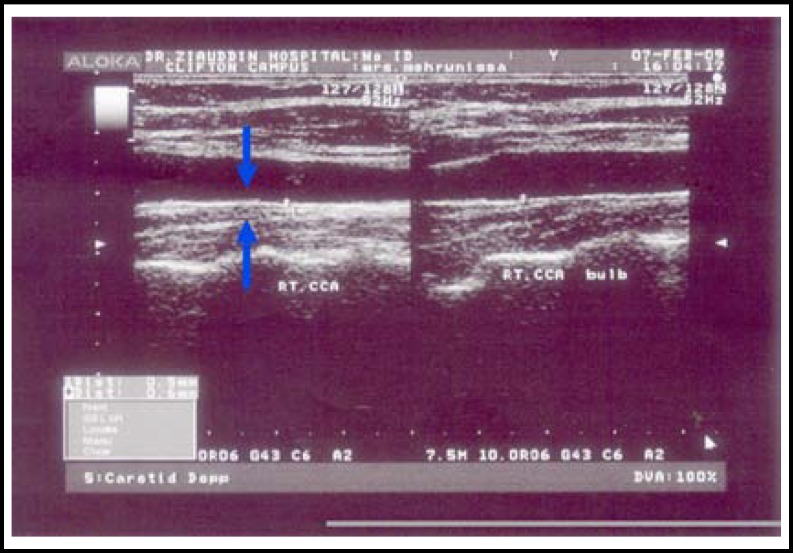
CIMT right common carotid artery.

**Fig.1(B):**
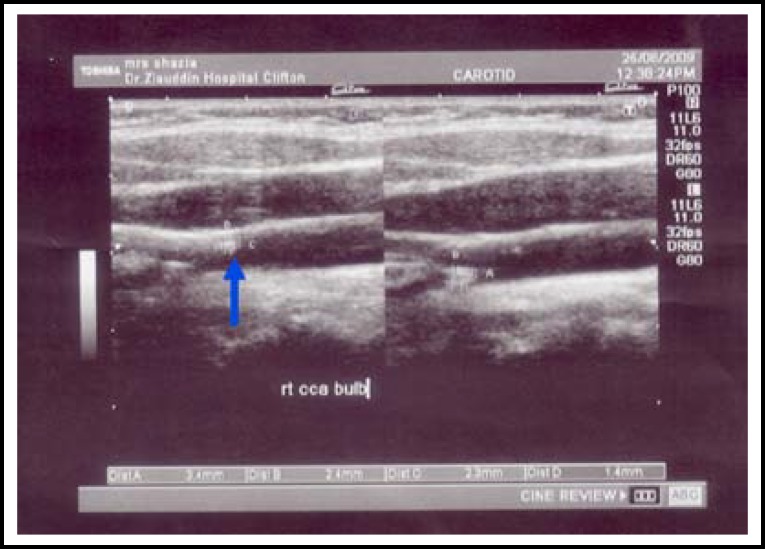
CIMT right bifurcation of carotid artery (plaques seen).

In this study the CIMT was measured on the far wall. Study by Agewall et al^21^, shows that CIMT should be measured preferably on the far wall. Carotid intima-media thickness values from the near wall are less reliable. It is possible to visualize plaques in the artery with ultrasound technique. Plaque is a focal structure encroaching into the arterial lumen of at least 0.5 mm or 50% of the surrounding CIMT. Several studies have shown a significant relationship between CIMT and cardiovascular risk. Also the number of cardiovascular risk factors correlation with CIMT. Cardiovascular diseases risks slow down as CIMT progression is reduced. This is most clearly shown when cholesterol and LDL-cholesterol levels are lowered. Increased CIMT and plaque predicts risk of stroke and acute myocardial infarction.^22^

In these patients having high LDL-C and increased carotid intima media thickness more significant at carotid bifurcation predicts an increased risk of early atherosclerosis.

## CONCLUSIONS

Increased carotid intima- media thickness in hypercholesterolemia. Carotid bifurcation has shown greater increase in intima-media thickness and plaques. Carotid- intima media thickness at bifurcation is most important site for measurement of IMT for determination of early atherosclerosis.
